# Composting: A biosecurity measure to maximize the benefit of broilers̕ litter

**DOI:** 10.5455/javar.2023.j699

**Published:** 2023-09-24

**Authors:** Samah Eid, Heba M. Hassan, Nayera M. Al-Atfeehy, Karim M. Selim, Amal S. A. El Oksh

**Affiliations:** 1 Bacteriology Department, Reference Laboratory for Quality Control on Poultry Production (RLQP), Animal Health Research Institute (AHRI), Agriculture Research Center (ARC), Egypt; 2 Virology Department, RLQP, AHRI, ARC, Egypt; 3 Biotechnology Department, RLQP Sharkia Branch, AHRI, ARC, Egypt

**Keywords:** *Clostridium*, compost, gene expression, Newcastle, *Salmonella*

## Abstract

**Objective:**

This study was conducted to evaluate the effect of composting on the count of *Salmonella* spp., *Clostridium perfringens*, and New Castle virus (NDV) isolated from broilers’ litter. Moreover, to verify the impact of compost thermal stress on the expression of thermal genes harbored in the isolated bacteria.

**Materials and Methods:**

The prevalence of enteric aerobic and anaerobic infections by *Salmonella* spp., *C. perfringens*, and viral infections by NDV were investigated in litter samples collected from 100 broiler flocks by conventional methods and polymerase chain reaction.

**Results:**

The samples were positive for *Salmonella* spp., *C. perfringens*, and NDV, with prevalence rates of 60%, 55%, and 30%, respectively. An experiment to study the effect of compost on the microbiological quality of litter was applied using five compost heaps with an initial average count of *Salmonella typhimurium* (3.2 × 10^5^CFU CFU/gm), *C. perfringens* (6.4 × 10^5^ CFU/gm), and an average titer NDV (10^5.5^ embryo infectious dose_50_/gm). The microbiological count of heaps after 15 days of composting revealed a reduction in the count of *S*. *typhimurium* and *C. perfringens* by 4 log_10_ CFU/gm and 3 log_10_ CFU/gm, respectively. Moreover, the hemagglutinating test revealed no detection of NDV after 15 days of composting. A high degree of downregulation of expression of the thermal genes, *dna*K in *S. typhimurium* isolates and *cpe* gene in *C. perfringens* isolates, was detected by quantitative reverse transcription PCR.

**Conclusion:**

The reduction of pathogen counts, the simplicity, and the low cost associated with composting for only 15 days advocate the recommendation for raising awareness of composting as a routine biosecurity measure to prevent the spreading of infection and promote its safe use in agribusiness.

## Introduction

The poultry industry is one of the most significant and rapidly expanding worldwide agribusinesses. Moreover, one of the major global food security pillars. Egypt has a huge poultry industry involving about 3 million working hands, 100 billion LE investment, 1.4 billion annual broiler bird production, 13 billion annual table egg production, and 25,000 farms, in addition to approximately 350 million birds in households.

Poultry litter is defined as a mixture of feathers, initial bedding material, dung, feed, and chicken waste [1]. The poultry industry is recognized as a leading and rapidly expanding agribusiness globally, playing a vital role in safeguarding food security worldwide [2]. In chicken houses, various litter materials made from agricultural wastes are employed, such as recycled paper, straw, wood shavings, rice hulls, and maize cobs. The litter must be conveniently accessible, nontoxic, porous, capable of absorbing much moisture, and affordable [3].

Many serious problems for the food industry and public health authorities are due to *Salmonella*; a Gram-negative foodborne bacterium that causes widespread contamination and illness [4]. *Clostridium *spp*.* is an endospore-forming bacteria associated with mild diarrhea symptoms [5]. In addition, Newcastle viral disease, caused by an avian paramyxovirus (APMV), poses significant economic consequences for the poultry industry and has been observed in various bird species, with domestic chickens being particularly affected [6].

Using litter repeatedly is a widely acknowledged practice in contemporary commercial chicken farming for several reasons: cost-effectiveness in production, limited availability of litter sources, environmental sustainability, and the difficulties in managing and disposing of litter. Reusing litter offers both environmental advantages and diverse economic benefits for the chicken industry, such as decreased expenses on bedding material, reduced transmission of diseases, improved quality of bedding material, and the option to utilize it as fertilizer [7].

Implementing a window of 3 to 5 days for in-house composting between flocks can be a highly effective approach to decreasing microbial and viral loads. By achieving temperatures of 50°C or higher during composting, it becomes possible to significantly reduce or eliminate bacterial counts, mitigate most viral infections, and ultimately enhance bird performance [8].

As the poultry industry has grown worldwide, the volume of poultry litter has increased significantly, leading to concerns among poultry breeders regarding its proper utilization and safe disposal. Due to its abundant plant nutrients, chicken litter is considered a highly valuable organic resource for fertilization purposes [9].

Composting is a natural biological breakdown process that occurs in an aerobic, thermophilic environment. It may be used for day-to-day control of farm mortalities as well as carcass disposal during animal disease outbreaks [10].

Nevertheless, litter recycling encounters various challenges, particularly in Egypt, including the spread of diseases due to bacterial and viral infections, limited knowledge and information, and its status as a relatively new practice. Unfortunately, academics have not given sufficient attention to this aspect, resulting in a scarcity of Egyptian studies in this specific field [11].

The transcriptomic responses of bacteria to thermal exposure of 55°C and the identification of differentially expressed genes associated with heat shock after heat treatment remain largely unexplored and poorly understood [12]. The researchers investigated the bacterial growth response, gene expression responses, and transcriptomic analysis of heat stress responses at 42°C as being representative of conditions associated with live poultry body temperature. Moreover, there is limited knowledge concerning the effects of higher intermediate nonlethal temperatures that bacteria might encounter during postharvest processing [13].

The goal of this study was to estimate the prevalence of anaerobic bacteria, enteric aerobic bacteria, and viral infections in broilers’ litter, such as *Clostridium perfringens*, *Salmonella* spp., and New Castle virus (NDV). Our work also aimed to evaluate the effect of compost on improving the microbiological quality of litter, as demonstrated by the reduction of titer per gram for each of the studied pathogens. Moreover, to verify the impact of thermal stress caused by compost on the expression of thermal genes carried in the studied bacterial species.

## Materials and Methods

### Sample collection

Litter samples (*n = *100), each in the form of a bag of 50 kg weight, were collected from each of 100 small open-system boiler farms with a history of enteric manifestations and symptoms of *Salmonella* spp., *Clostridium *spp*.*, and NDV infection in Sharkia governorate. Litter bags were subjected to bacteriological examination and polymerase chain reaction (PCR) detection of NDV at reference lab of quality control of poultry production (RLQP), Sharkia Laboratory.

### Detection of studied pathogens

Isolation and identification of *Salmonella* spp.: Samples were examined according to ISO/IEC.ISO 6579-1:2017-AMD 2020 [14]. Cultures containing presumed colonies were securely transported in leakproof cold containers with proper bio-security measures to Animal Health Research Institute (AHRI) for serotyping. The serotyping process utilized *Salmonella* antiserum (Denka Seiken Co., Japan) as described by Grimont and Weill [15].

Isolation of *Clostridium* spp.: According to Fancher et al. [16], *Clostridium *spp. was isolated from examined samples; microscopical examination was used for suspected colonies as Gram-positive sporulated former rods; and biochemical examination of *C. perfringens* isolates was applied according to Shen and Chen [17]. Confirmed pure isolates of *Salmonella typhimurium* and *C. perfringens* were randomly selected among the isolates for use in the composting experiment.

### Detection of NDV by quantitative reverse transcription PCR (qRT-PCR)

Each sample underwent RNA extraction using the QIAamp viral RNA Mini kit (Qiagen, Germany, GmbH). The sample suspension (140 µl) was mixed with carrier RNA (5.6 µl) and AVL lysis buffer (560 µl) before being incubated at room temperature for 10 min. Following incubation, 100% ethanol (560 µl) was added to the lysate, and each sample was washed and centrifuged following the manufacturer’s instructions.

### VndF gene oligonucleotide

The primer and probe were listed in Table 1, provided by Metabion (Germany). The DNA amplification process was conducted in a final volume of 25 µl, comprising an RNA template (5 µl), QuantiTect Probe RT-PCR Master Mix (12.5 µl), PCR-grade water (6.625 µl), 50 pmol concentration of each primer (0.25 µl), 30 pmol concentration of each probe (0.125 µl), and QuantiTect RT Mix (0.25 µl). Reverse transcription occurred at 50°C for 30 min, followed by primary denaturation at 94°C for 15 min. Subsequently, 40 cycles were performed, involving denaturation at 94°C for 15 sec, annealing at 52°C for 30 sec, and extension at 72°C for 10 sec in the Stratagene MX3005P machine. Litter samples that were PCR-positive for NDV were safely transported to the RLQP main laboratory for isolation and titration of NDV for use in the compost experiment.

### Molecular confirmation of bacteria isolates

The QIAamp DNA Mini Kit (Qiagen, Germany, GmbH) was used for DNA extraction from different samples; oligonucleotide primers are demonstrated in Table 2. A PCR reaction (25 µl) was utilized for the amplification process, which included PCR-grade water (4.5 µl), Emerald Amp Max PCR Master Mix (Emerald, Japan) (12.5 µl), 20 pmol of each primer (1 µl), and an analyzed DNA template extract (6 µl). The PCR thermal profile was performed as follows: primary denaturation at 94°C for 5 min, amplification for 35 cycles, secondary denaturation at 94°C for 30 sec, annealing at 55°C for 40 sec, extraction at 72°C for 45 sec, and final extraction at 72°C for 10 min. By electrophoresis, PCR products were separated.

### Compost experiment

**Figure 1.**
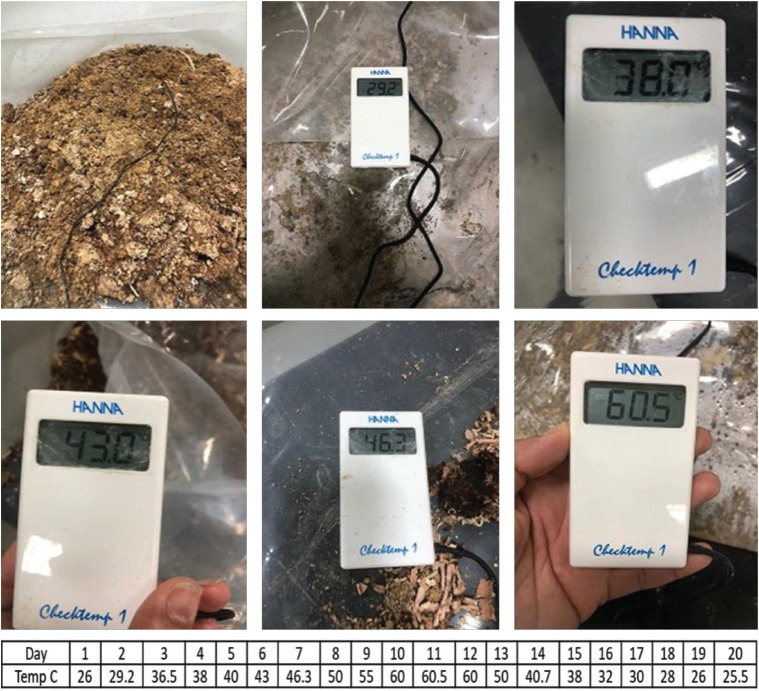
A compost heap of 1 m length *1 m width *90 cm height, was made from SPF broilers´ litter and sawdust, the digital thermohygrometer was used to monitor temperature for 20 days.

Composting was applied in the Experimental Animal Center—biosafety level 3 (BSL3) facility of the Animal Health Research Institute. Test bacterial isolates were adjusted in PBS against a 0.5 McFarland standard equivalent to 1.5 × 10^8^ CFU/ml for *S. typhimurium *and a 1 McFarland standard equivalent of 3 × 10^8^ CFU/ml for *C. perfringens*. The NDV strain preparation was adjusted to 10^5.5^ embryo infectious dose (EID) 50/ml allantoic fluid (GenBank accession no. MZ409479, Egypt-NDV-RLQP-2021). Preparations of test pathogens were evenly mixed with litter collected from specified pathogen free (SPF) chickens. The experiment involved five compost heaps, each of 1-m length, 1-m width, and 90 cm height. Experimentally infected litter was mixed with sawdust to give carbon/nitrogen ratios of 25:1. Water was added to wet the compost without excess to adjust the moisture percentage to approximately 65%. Temperature and moisture were monitored by a calibrated thermohygrometer by introducing its probe to the core of the heaps, which routinely flipped over and mixed to allow aeration [18] (Figure 1).

**Figure 2.**
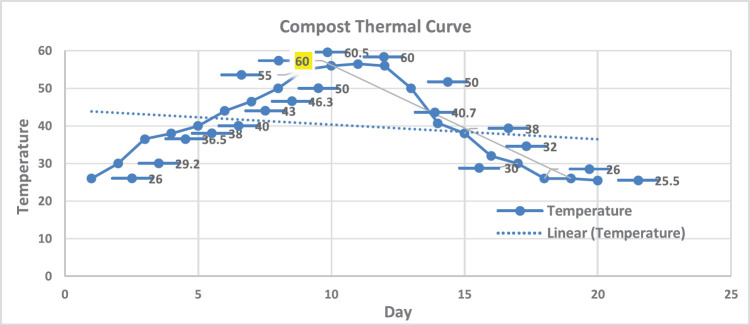
Compost temperature curve: phase 1, mesophilic temperature <40°C at days 4 and 5, phase 2; thermophilic phase >40°C from days 5 to 14, the highest temperature 60.5°C was recorded at day 11, mesophilic phase started from day 15, and drop-down cooling from day 17 till the end of the experiment at day 20.

The temperature of compost heaps was monitored daily during the 20 days of the experiment duration; water content was adjusted according to the need to maintain compost consistency; and litter sawdust was also added as needed to maintain the consistency of the compost mixture and keep elevation. The temperature started to elevate on the second day and reached a peak of 60.5°C on day 11 (Figure 2).

On the 15th day of the experiment, as the temperature started to drop below 40°C, samples from each heap were collected and tested for *S. typhimurium* and *C. perfringens* counts per gram and NDV titers per gram. The temperature monitor continued in the cooling period until the temperature reached the ambient room temperature at the end of the experiment on the 20th day.

**Table 1.** NDV primers and probes.

Virus	Sequence of primer/probe (5’-3’)	Reference
*Vnd* F^1^	F+4839) TCCGGAGGATACAAGGGTCT)	[24]
	(F-4939) AGCTGTTGCAACCCCAAG	
	F+4894) [FAM]AAGCGTTTCTGTCTCCTTCCTCCA[TAMRA])	

*Vnd* F^1^: NDV detection gene.

**Table 2.** Target genes, primer sequences, and amplicon sizes of different bacterial species.

Test target	Target gene	Sequences of primers (5’-3’)	Amplified segment (bp)	Reference
*Salmonella* spp.	*inv*A^1^	GTGAAATTATCGCCACGTTCGGGCAA	284	[25]
		TCATCGCACCGTCAAAGGAACC		
*Clostridium *spp*.*	*16S rRNA* ^2^	AAAGATGGCATCATCATTCAAC	360	[26]
		TACCGTCATTATCTTCCCCAAA		

*inv*A^1^: invasion of the host epithelial cells (Conserved virulence gene),* 16S r*RNA^2^:* Clostridium spp.* Conserved gene.

Qualitative indicators for good hygiene and maintenance of the aerobic condition of compost were monitored throughout the experiment, represented by the absence of offensive odor and flies. The experiment was applied in the BSL3 laboratory animal facility at AHRI to ensure containment and prevent infection spreading to the external environment.

### Microbiological quality of composted litter

*Salmonella typhimurium* count: A sample from each of the 5 heaps was collected and diluted 10-fold serially in sterile saline. On *Salmonella Shigella* agar, 0.1 ml of each dilution was spotted, and it was incubated aerobically for 24 h at 37°C. The microbial count was expressed as log_10 _CFU/gm of the sample [19].

The count of *C. perfringens* was determined by collecting samples from each of the 5 heaps and subjecting them to a 10-fold dilution in sterile saline. The initial dilution was incubated for 45 min in a water bath at 75°C, and then further 10-fold serial dilutions were made in sterile saline. To examine the growth and count colonies, 0.1 ml of each diluted sample was plated onto blood agar and incubated anaerobically at 37°C overnight [20].

### Titration of NDV in the composted litter

The concentration of NDV in a suspension is expressed as a titer. Titration is used to determine the infectivity titer of NDV suspension, which is the number of infectious units of virus per unit volume, often expressed per ml. The 50% EID_50_ unit is used to evaluate the virulence of NDV.

A series of 10-fold dilutions are performed on a suspension of 1 gm of infected litter with NDV to evaluate the infectivity titer of the suspension. Each dilution contains an inoculation of five embryonic eggs. The hemagglutinating (HA) test is used to investigate if the virus has infected and multiplied in each egg after 3–5 days of incubation, which is used to calculate the infectivity titer [21].

The mathematical method developed by Ramakrishnan [22] is used to determine the dilution of the virus suspension being tested and create the endpoint, the endpoint containing one unit of infectivity (1 EID_50_). The infectivity titer is then calculated using this dilution.

### Thermal gene expression of studied bacterial species

Tris EDTA buffer (200 µl) and Thermo Fisher Scientific, GmbH, Germany’s lysozyme (1 mg/ml) were added to the bacteria pellet, and then the steps were completed according to Qiagen, Germany, GmbH’s QIAamp RNeasy Mini kit for extracting RNA from bacteria.

The genes were chosen according to the information available, and the primers were evaluated by SYBR green rt-PCR using the Stratagene MX3005P machine, which utilized a 25 μl reaction as a one-step reaction of QuantiTect SYBR Green PCR Master Mix (Qiagen, Germany, GmbH) (12.5 μl), RevertAid Reverse Transcriptase (0.25 μl), Thermo Fisher Scientific, GmbH, Germany (200 μl), different primer (0.5 μl), PCR-grade water (8.25 μl), and purified RNA (3 μl). The thermal profile was created by performing reverse transcription at 50°C for 30 min, primary denaturation at 94°C for 15 min, amplification (40 cycles), secondary denaturation at 94°C for 15 sec, extension at 72°C for 30 sec, and a dissociation curve at 1 cycle. The annealing temperature is shown in Table 3, with the secondary and final denaturations at (94°C/min) and (94°C/min), respectively.

A related bacterial housekeeping gene was used to normalize the relative expression of each thermal gene. By the ΔΔ cycle threshold (CT) method, the untreated control sample was used to compare the CT value of each one to estimate the relative quantitation of gene expression on RNA templates of different samples [23].

## Results

### Prevalence of studied pathogens in the litter

Litter samples were subjected to conventional bacteriological techniques to identify *Salmonella* spp. and *C. perfringens.* Suspected colonies were further confirmed using PCR to detect relevant conserved genes. The findings indicated that 40 out of 100 (40%) litter samples were positive for *Salmonella* spp., while 35 out of 100 (35%) samples tested positive for *C. perfringens*. In addition, 10 out of 100 (10%) samples showed positive results for NDV through qRT-PCR. Moreover, the results indicated that 20 out of 100 (20%) litter samples exhibited mixed infections involving all three pathogens studied, as shown in Table 4.

**Table 3.** Target genes, primers sequences, and annealing conditions of SYBR green rt-PCR.

Target bacteria	The function of the target gene	Primer sequences (5’-3’)	Amplification (40 cycles) Annealing °C/sec	Dissociation curve (1 cycle) Annealing °C/min	Reference
*Salmonella *spp*.*	*16S r*RNA*^1^*	CAGAAGAAGCACCGGCTAACTC	60/30	60/1	[27]
		GCGCTTTACGCCCAGTAATT			
	*dna*K*^2^*	CGCTTCCAGGACGAAGAAGT	55/30	55/1	[28]
		CGAGGTCGTAAACCGCGATA			
*Clostridium *spp*.*	*16S r*RNA*^1^*	AAAGATGGCATCATCATTCAAC	53/30	53/1	[29]
		TACCGTCATTATCTTCCCCAAA			
	*cpe^3^*	ACATCTGCAGATAGCTTAGGAAAT	55/30	55/1	[30]
		CCAGTAGCTGTAATTGTTAAGTGT			

*16S r*RNA*^1^* *: *Housekeeping gene*, dna*K*^2^* *:* Chaperone protein gene*, cpe^3^* *:* Enterotoxin gene.

### Serotyping of Salmonella isolates

According to Table 5, serotyping of *Salmonella* isolates identified five serotypes, including *S*. *typhymurium* and *S. enteritidis* (15/60 each), *Salmonella Heidelberg* (10/60), *Salmonella Kentucky* (5/60), and *S*. *Emek* (5/60).

### Count of Salmonella spp. and Clostridium spp. in composted litter

The composting experiment investigated the survival and thermal resistance of *S. typhimurium *and *C. perfringens* in five composted litter heaps. The results revealed a reduction in the count by an average of 4 log_10_ CFU/gm in *S. typhimurium* and a reduction in the count by an average of 3 log_10_ CFU/gm in *C. perfringens *(Table 6).

### Gene expression analysis of thermal genes in S. typhimurium and C. perfringens

**Figure 3.**
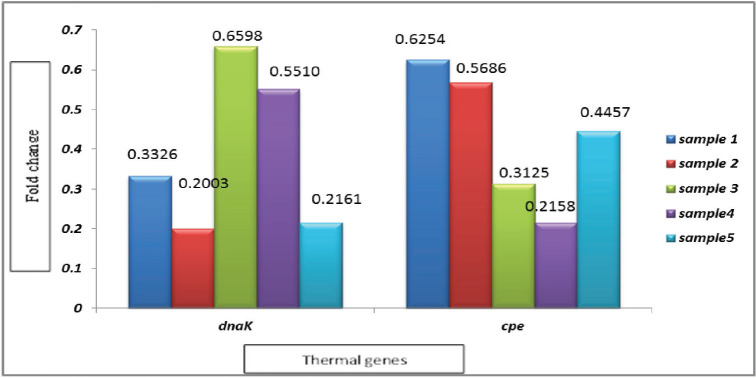
Effect of temperature treatment on thermal genes expression of *S. typhimurium* and *C. perfrngens *isolates*.*

Gene expression analysis for thermal genes (chaperone protein*, dna*K gene in *S*. *typhimurium* and enterotoxin,* cpe* gene in *C. perfringens* on the RNA level of five compost samples was examined. Thermal gene expression of isolated microbial RNA demonstrated varying degrees of thermal gene downregulation after compost treatment compared to untreated isolates before composting. Degrees of downregulation ranged from 0.20- to 0.66-fold for the *dna*K gene of *S. typhimurium *and from 0.22- to 0.63-fold for the *cpe* gene of *C. perfringens *(Figure 3).

**Table 4.** Prevalence of tested pathogens recovered from litter broiler flock.

Type of infection	Tested pathogens	Prevalence (%)
Single infection	*Salmonella *spp.	40/100 (40)
	*Clostridium *spp.	35/100 (35)
	NDV	10/100 (10)
Mixed infection	*Salmonella* spp. *Clostridium* spp. NDV	20/100 (20)

**Table 5.** *Salmonella* spp. Serotyping.

Serovars	Antigenic structure		Number of isolates
	O	H	
*Salmonella typhymurium*	1,4,5,12	i : 1,2	15/60
*Salmonella Kentucky*	8,20	i : Z6	5/60
*Salmonella Enteritidis*	1,9,12	g,m : -	15/60
*Salmonella heidelberg*	1,4,5,12 r : 1,2		10/60
*Salmonella emek*	4,12 i : Z6		5/60
Total number of isolates			60/60

### Titration result of NDV suspension in composted samples

**Figure 4.**
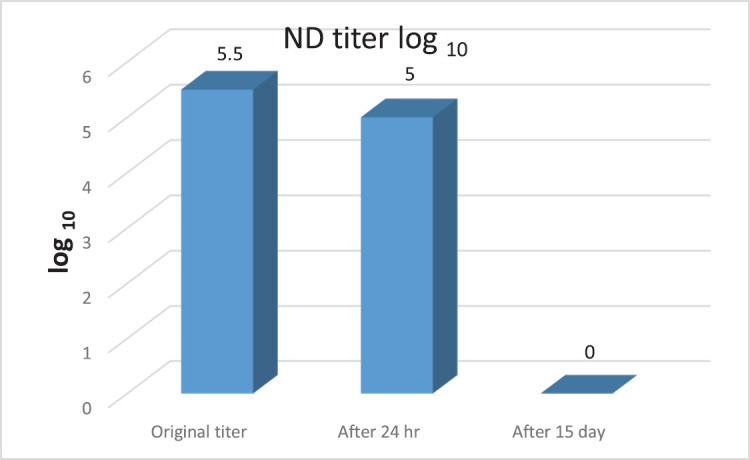
Effect of NDV titer in composted litter.

The HA test results of five inoculated eggs on allantoic fluid are listed in Table 7, the percentage of infection was calculated from this data. The titer of NDV from litter is 10^5.5^, after 24 h is 10^5^ in five composted litter heaps; and after 15 days, it is near zero as the virus is not detected by HA or a dead embryo, according to Ramakrishnan [22], as shown in Figure 4.

## Discussion

*Salmonella, Clostridium* species, and NDV are among the primary bacterial and viral infections that significantly impact the poultry industry. These infections lead to substantial economic losses in poultry production due to increased mortality rates, incidences of illness, and high expenses for preventive measures, therapeutic medications, and vaccinations [31,32].

*Salmonella* spp. and other pathogens were found in the environment, and in many instances of the breeding cycle phase, these pathogens were extended many times in the environment, which is commonly found in poultry litter [33].

In our study, 40% of litter samples were positive for *Salmonella* spp. In addition, 20% of the samples exhibited mixed infections of *Salmonella* spp., *C. perfringens*, and NDV. The presence of *Salmonella* spp. was confirmed through PCR, specifically targeting the conserved *inv*A gene of the genus *Salmonella*, aligning with the findings reported by Jibril et al. [34], who detected a prevalence rate of *Salmonella* spp. in poultry litter (47.9%). The lower prevalence rate of *Salmonella* spp. (3%) in poultry litter was recorded by Dahshan et al. [35].

The identified serovars posed a significant risk to public health as they were considered a warning that zoonotic pathogens from poultry farms might reach the food chain [33]. Serotyping of *Salmonella* isolates revealed that *S.* *typhimurium* and *S.* *enteritidis* represented the serotypes with the highest detection rate of 15/60 isolates each, *Salmonella heildelberg* with a detection rate of 10/60 isolates, and *S.* *Kentucky* and *S.* *Emek* were the least detected serotypes at 5/60 each. In the same regard, Kaoud et al. [33] collected poultry litter from several Egyptian governorates, including isolated *S*. *typhimurium* and *S.* *enteritidis* with a prevalence rate of 11.67% for each, while *S*. *heildelberg* (15.2%) was linked to substantial economic losses due to a high mortality rate (4%–50%), weight loss, and lower egg production. In the same instant, Soliman et al. [32] recorded that 46.5% of the examined poultry litter was positive for *S*. *typhimurium*. 

*Clostridium perfringens* in poultry causes gangrenous dermatitis, which clinically includes ataxia, fever, and subcutaneous soft tissue damage, whereas necrotic enteritis can result in sudden death, diarrhea, dehydration, and anorexia [36]. In this study,* C. perfringens *isolation from litter samples revealed that 35% were positive. Moreover, 20% of examined samples were positive for mixed infection with *C. perfringens, Salmonella,* and NDV; presumptive *C. perfringens* were confirmed by PCR detection of the conserved (*16S* *rRNA)* gene. This finding was in accordance with Abougabal [18], who recorded that 40% of poultry litter samples were positive for* C. perfringens.* Abd-Elall and Maysa [37] detected *C. perfringens* from broiler litter with a prevalence rate of 38.7% in Sharkia province, Egypt.

The virulent NDV is an infectious illness of poultry with ubiquitous symptoms, such as nervous manifestations, acute respiratory infections, depressed symptoms, and diarrhea. The severity of the disease is influenced by the host’s susceptibility and the virus’s virulence, which may result in trade restrictions [38].

qRT-PCR results revealed NDV in 10% of litter samples; PCR results also revealed the positivity of 20% of litter samples for NDV in addition to *Salmonella* spp. and *C. perfringens*. These outcomes supported the findings of Nurzijah et al. [39], who recorded a high prevalence rate (45%) of NDV in poultry flocks with mixed infections.

Properly handled poultry manure is the most valuable among all manures produced by poultry flocks [32]. The practice of utilizing treated litter from different areas is widely adopted in poultry production due to its cost-effectiveness and the significant benefits it offers in reducing bacterial and viral diseases, which are highly recommended in various regions across the globe [18]. Seasonal variation impacts the intensity, prevalence, and distribution of poultry diseases, which is considered a major limitation of poultry production. Diseases and an unfavorable climate are regarded as key restrictions for poultry production and are associated with high mortality rates in young birds [40]. In addition to the high pH and moisture content of untreated litter in broiler farms, it is thought to be one of the best mediums for the growth and transmission of pathogens [32].

**Table 6.** Bacterial count recovery from composted litter heaps.

Replication	Composted samples (log_10_ CFU/gm)			
	*Salmonella typhimurium* count-CFU/gm		*Clostridium perfringens* count-CFU/gm	
	Initial count on 1st day	Count on 15th day	Initial count on 1st day	Count after 15th day
CH1^*^	3 × 10^5^	1.2 × 10	7 × 10^5^	2.9 × 10^2^
CH2	5 × 10^5^	2 × 10	6 × 10^5^	3.2 × 10^2^
CH3	4 × 10^5^	4 × 10	8 × 10^5^	1.6 × 10^2^
CH4	2 × 10^5^	2.3 × 10	5 × 10^5^	1.4 × 10^2^
CH5	2 × 10^5^	2.3 × 10	6 × 10^5^	4.1 × 10^2^
Average	3.2 × 10^5^	2.4 × 10	6.4 × 10^5^	264
SD	1.3 × 10^5^	2.3 × 10	1.1 × 10^5^	1.1 × 10^2^
*p*-value	0.03%		0.0001%	
Significance level	99.97%		100%	

CH^*^: Compost heap, *p*-value ≥ 5% = nonsignificant, *p*-value<5%.

**Table 7.** Extract Ramakrishnan’s working sheet on 15th day.

Inoculum dilution	No. of infected eggs (HA +Ve)		No. of noninfected eggs (HA -Ve)		Accumulated numbers	
					Infected (A)	Noninfected (B)
10^−2^	1	↑	4	-	1	4
10^−3^	0	↑	5	-	0	9
10^−4^	0	↑	5	-	0	14
10^−5^	0	-	5	-	0	19
10^−6^	0	↑	5	-	0	24

In the present study, feedback was collected from field veterinarians and farmers about their knowledge of composting and the reasons for the limited domestic practice of in-house composting despite much organized awareness and training for composting over the last two decades. Interestingly, an agreement on the reason behind the reluctance for in-house compost was associated with the long period believed to be required for effective composting (4 weeks), which imposes a long stop period in between breeding cycles. This feedback was considered by the farmers to be a potential economic loss. Therefore, the current experiment was designed to investigate the impact of compost on the microbiological quality of litter during a relatively shorter composting period (20 days).

In our research, we conducted a composting experiment to examine the survival of *Salmonella spp., C. perfringens*, and NDV in composted litter under ambient temperatures exceeding 60°C. During the composting process, the temperature reached its peak of 60.5°C on the 11th day and was maintained at 60°C–60.5°C for 72 h under aerobic conditions. Abougabal [18] revealed that temperatures were measured on the surface and interior of the litter heaps at each sample. Although these temperatures were varied (26°C *vs.* 56°C, respectively), the mean temperatures did not vary considerably over time, as did Abouelenien et al. [41], who incubated chicken litter in an Egypt farm (cage layer system) at 25°C versus 55°C. Das et al. [42] recorded a reduction in bacteria count by raising the temperature to 60.5°C during composting of hatchery wastes in poultry litter. The process of composting typically consists of two phases: a curing (or maturation) phase and a heating (or developing) phase during which thermophilic temperatures (>50°C), high oxygen consumption, and a rapid reduction in biodegradable solids occur [43,44].

In our study, we observed a significant reduction in *S. typhimurium* count, amounting to (4 log_10_ CFU/gm), and a reduction in the count of *C. perfringens,* amounting to (3 log_10_ CFU/gm). *Salmonella* and *Clostridium* spp. levels can be significantly reduced by performing in-house composting while they are eliminated from composted samples, but they are still recoverable in uncomposted samples [45]. These findings are consistent with the results reported by Beasley-Hall et al. [12] and Mesas et al. [46], who detected a reduction in the count of *Salmonella* spp. and *C. perfringens* (3 log_10_ CFU/gm) for each after composting, while Ahmed et al. [47] recorded a significant reduction of *Salmonella spp*. count after composting from (3.4 × 10^2^ to 1.0 × 10^2^ CFU/gm) and *Clostridium spp*. decrease from (3.8 × 10^4^ to 1.0 × 10^4^ CFU/gm) at the end of compost.

In the pursuit of biological understanding, measurements of gene expression utilize all available sequencing information for experimental design and data analysis [48]. In the present study, the thermal resistance of pathogens against heat treatment was studied by investigating the gene expression of two heat shock genes, the *dna*K gene in isolated *Salmonella* spp. and the *cpe *gene in* C*. *perfringens* isolates, by qRT-PCR. A high degree of bacterial heat shock gene downregulation was detected. In the same regard, Ehuwa et al. [4] concluded that the heat shock gene (*dnaK*) and chaperone protein were repressed in DNA replication by heat treatment, which led to the synergism of heat shock genes in *Salmonella* spp. by membrane damage.

Our study revealed that heat treatment had a notable impact on the gene expression levels of isolates, as the RNA expression level in the treated isolates differed significantly from that of the untreated or negative controls. This finding is consistent with the results reported by Chen et al. [49], who also investigated the effect of heat treatment on gene expression in various isolates before and after treatment.

To assess the expression of the enterotoxin gene (*cpe*), we employed qRT-PCR, and the results demonstrated that heat treatment effectively reduced the growth of germinated spores of *C. perfringens*. These findings align with those of Kawarizadeh et al. [50], who observed a reduction in the expression of the *cpe* gene in *C. perfringens* in treated broiler litter.

In the current analysis, titration of NDV by HA in composted litter heaps showed positive hemagglutination activity in titrated NDV-contaminated samples after 24 h (10^5 ^EID_50_/gm), and the positive samples were fresher than the samples from compost. A negative HA test was recovered after 15 days, as mentioned in Figure 3. Due to the high temperatures achieved within the compost (>60°C) from day to day, the same results agreed with those of Benson et al*. *[51] and Guan et al. [52].

NDV was quickly inactivated when compost temperatures were >60°C; on the other side, when embryonated chicken eggs (ECEs) were inoculated with NDV, there were no signs of damage, and the virus was not detected in those samples until the study concluded on the 20th day. Notably, it was observed that the degradation of the virus load was faster in used litter compared to other samples, possibly due to higher microbial activity in that particular specimen. Our study’s findings are consistent with those of Mo et al. [53], who discussed the thermal stability of APMV-1 in poultry litter. Virus inactivation was assessed at temperatures ranging from 10°C to 43.3°C at 5.5°C intervals. Wood shavings-based poultry litter was given a high titer of virus of about (10^8^ EID_50_), with an evaluation of the moisture level in the litter. Samples were collected at different time intervals, and infectious viruses were titrated in ECEs. Infectious viruses could not be detected after 2–7 days at high temperatures (37.8°C to 43.3°C), whereas at lower temperatures (10°C to 21.1°C), it took up to 112 days for viruses to decrease to undetectable levels.

Feedback from field veterinarians in Sharkia governorates revealed that composting is not commonly applied in broiler farms in Sharkia; the owners are usually not willing to do composting between broiler breeding cycles to allow rapid housing of subsequent breeding cycles and avoid the long stop period (4 weeks) required for composting. The owners prefer to sell the untreated litter, which generates 4 m^3^/1,000 birds with an average price of 500 LE/m^3^. The untreated litter is sold to subcontractors, including aquaculture farms in Kafr Sheikh and mushroom farms in North Sinai. This feedback signifies the high risk of transporting untreated litter and spreading infections among farms between governorates. Moreover, highlights the presence of a great opportunity to promote a short-term (15 days or 2 weeks) composting approach that was proven to produce a significant reduction of pathogen titers in litter, reducing the potential risk of spreading the infection and allowing for safe movement of litter. Moreover, improves the economic value of composted litter versus untreated litter, thus adding economic value to farm owners.

By reviewing the proceeded study, weak points were demonstrated in the importance of maintaining a high temperature under aerobic conditions for 5 to 6 days to obtain successful compost, which requires awareness of the farmers for the alarming indicators of anaerobic conditions represented in flies infestation and the offensive odd smell, as well as to avoid the drop in the high temperature below 40°C. Thus, continuous monitoring is required throughout the 15 days to ensure aeration by mixing, maintain the C:N ratio by adding sawdust or litter, and adjust the humidity by wetting as required.

## Conclusion

The promising results of the study revealed that proper composting of broilers’ litter for 15 days resulted in a significant reduction in the count of *S. typhimurium, C. perfringens,* and the titer of the NDV. Thus, advocates promote composting as a simple, low-cost, and effective biosecurity strategy to reduce the spread of pathogenic bacteria and viruses in poultry farms.

## List of Abbreviations

AHRI, Animal Health Research Institute; APMV-1, Avian paramyxovirus-1; BSL3, Experimental Animal Center Biosafety level 3; *C*., *Clostridium*; CT, cycle threshold; ECEs, embryonated chicken eggs; EID_50_, embryo infected dose 50; HA, hemagglutinating test; NDV, New Castle virus; PCR, polymerase chain reaction; qRT-PCR, quantitative reverse transcription PCR; RLQP, reference lab of quality control of poultry production; S., *Salmonella*; SD, standard deviation; SPF, specified pathogen free.
